# Position-Space-Based Design of a Symmetric Spatial Translational Compliant Mechanism for Micro-/Nano-Manipulation

**DOI:** 10.3390/mi9040189

**Published:** 2018-04-17

**Authors:** Haiyang Li, Guangbo Hao

**Affiliations:** School of Engineering-Electrical and Electronic Engineering, University College Cork, T12 K8AF Cork, Ireland; haiyang.li@umail.ucc.ie

**Keywords:** compliant mechanism, position space, analytical modelling, symmetric design, micro-/nano-manipulation

## Abstract

Symmetry enables excellent motion performance of compliant mechanisms, such as minimized parasitic motion, reduced cross-axis coupling, mitigated buckling, and decreased thermal sensitivity. However, most existing symmetric compliant mechanisms are heavily over-constrained due to the fact that they are usually obtained by directly adding over-constraints to the associated non-symmetric compliant mechanisms. Therefore, existing symmetric compliant mechanisms usually have relatively complex structures and relatively large actuation stiffness. This paper presents a position-space-based approach to the design of symmetric compliant mechanisms. Using this position-space-based approach, a non-symmetric compliant mechanism can be reconfigured into a symmetric compliant mechanism by rearranging the compliant modules and adding minimal over-constraints. A symmetric spatial translational compliant parallel mechanism (symmetric XYZ compliant parallel mechanism (CPM)) is designed using the position-space-based design approach in this paper. Furthermore, the actuation forces of the symmetric XYZ CPM are nonlinearly and analytically modelled, which are represented by the given primary translations and the geometrical parameters. The maximum difference, between the nonlinear analytical results and the nonlinear finite element analysis (FEA) results, is less than 2.58%. Additionally, a physical prototype of the symmetric XYZ CPM is fabricated, and the desirable motion characteristics such as minimized cross-axis coupling are also verified by FEA simulations and experimental testing.

## 1. Introduction

Compliant mechanisms transmit and transform displacements, forces, and energy using elastic deformation of their compliant members, leading to merits such as no backlash and no friction compared with their rigid-body counterparts [1,2,3,4,5,6]. They have been widely employed in many applications such as atomic force microscopy [7,8], nano-/micro-assembly [9,10], nano-/micro-positioning [11,12], nanostructures [13], microelectromechanical systems [14,15], soft robots [16], and multiplex optical switches [17]. However, the stiffness of a compliant member is neither zero in the degree of freedom (DOF) directions nor infinitely large in the degree of constraint (DOC) directions [18]. Consequently, compliant mechanisms, with a particular emphasis on distributed compliant mechanisms [19,20,21,22], often suffer from cross-axis couplings and parasitic motions [1,2,3,4,5,6] due to the nature of their deformation. The cross-axis couplings should be minimized for avoiding complex control, especially when output displacement sensors are not available [23]. The parasitic motions should be maximally reduced using different method such as optimizing the dimensions and improving the structures [24,25], since it is very hard to be compensated by the control systems.

A compliant mechanism can be regarded as a combination of rigid stages and compliant modules [26]. A compliant module is a compositional unit of a compliant mechanism (also known as a sub-compliant mechanism), which includes compliant members and their rigid links (RLs). Each compliant module in a DOF/DOC-specified compliant mechanism has many possible permitted positions, while the set of all the permitted positions is defined as the position space of the compliant module in the compliant mechanism [26,27,28]. If a compliant module in a compliant mechanism is rearranged to another possible permitted position within its position space, the DOF or DOC of the compliant mechanism remains unchanged [26,27,28].

**Figure 1:**
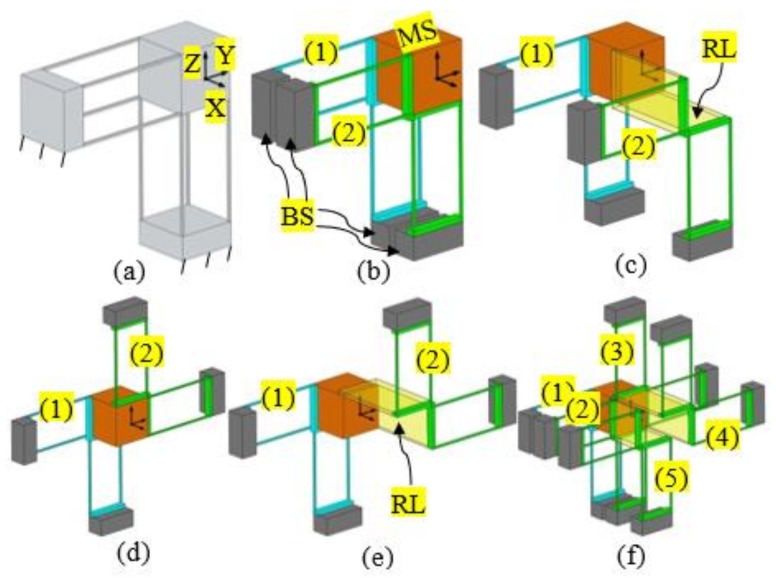
Position-space-based reconfiguration of a general one degree of freedom (1-DOF) translational compliant mechanism: (**a**) original 1-DOF translational compliant mechanism; (**b**) decomposition of the 1-DOF translational compliant mechanism; (**c**) change of geometrical dimension; (**d**) change of geometrical shape; (**e**) changes of both geometrical dimension and geometrical shape; and (**f**) addition of redundant compliant modules (MS: motion stage, BS: base stage, RL: rigid link, compliant modules are labelled by numbers (1) to (5)).

Changing the positions of the compliant modules in a compliant mechanism can change both the geometrical dimension and the geometrical shape of the compliant mechanism. For example, the original 1-DOF translational compliant mechanism [29], as shown in Figure 1a, can be decomposed into a motion stage (MS), four base stages (BSs), and two double-two-beam compliant modules (DTBCMs), as shown in Figure 1b. The two DTBCMs, with their BSs, can be moved to other permitted positions within their position spaces. The permitted positions of the two DTBCMs can be represented by the relative positions to the original MS, which can be derived based on the screw theory as explained in [26]. Note that the derivation of the position spaces is not detailed in this paper. When the positions of the two DTBCMs are changed, both the geometrical dimension and the geometrical shape are changed, as seen in Figure 1c–e. Figure 1c shows that the spanning size between the two DTBCMs is increased, via translating one of the DTBCMs along the X-axis and linking it to the MS using a RL. Figure 1d shows that the geometrical shape of the compliant mechanism is changed through rotating one of the DTBCMs about the X-axis at 180°. A change associated with both the geometrical dimension and the geometrical shape is illustrated in Figure 1e. Furthermore, from Figure 1f, it can be seen that a permitted position of a compliant module can also be a position to add a redundant copy (over constraint) of the compliant module. In Figure 1f, three redundant copies (labelled as numbers 3, 4, and 5) of the DTBCM are added to the 1-DOF translational compliant mechanism, which does not affect the DOF of the compliant mechanism.

In order to improve the motion performance, a compliant mechanism can be optimized into a symmetric compliant mechanism. It can be claimed that in comparison with a non-symmetric design (therefore, a simple configuration) the symmetric design enables better performance of compliant mechanisms such as minimized parasitic motion, reduced cross-axis coupling, mitigated buckling, and decreased thermal sensitivity [5,23,30,31]. However, existing symmetric compliant mechanisms are usually highly over-constrained (by directly adding equal redundant compliant modules), leading to very large actuation stiffness [5,18,23].

**Figure 2:**
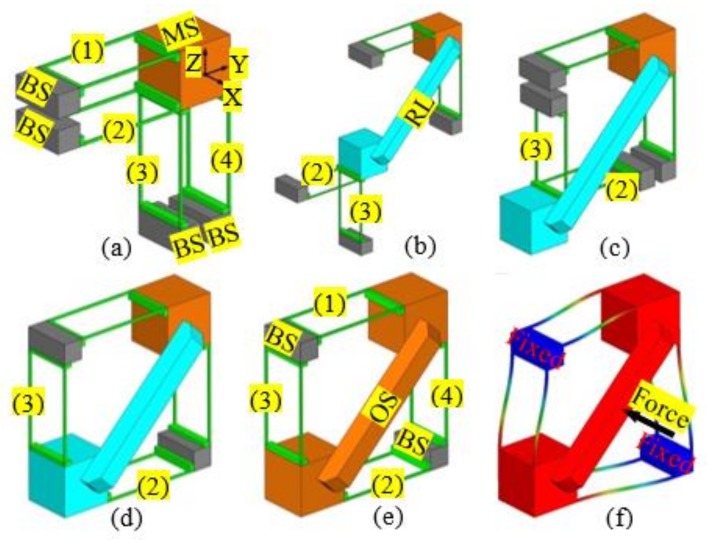
Position-space-based reconfiguration for generating a 1-DOF symmetric translational compliant mechanism: (**a**) decomposition of the 1-DOF translational compliant mechanism; (**b**) translations of compliant modules; (**c**) rotations of compliant modules; (**d**) further translations of compliant modules; (**e**) symmetric 1-DOF translational compliant mechanism; and (**f**) deformation of the 1-DOF translational compliant mechanism under an actuation force.

Because changing the positions of the compliant modules in a compliant mechanism can be used to change the geometrical shape of the compliant mechanism, it is possible to reconfigure a non-symmetric compliant mechanism into a symmetric compliant mechanism via rearranging the compliant modules and adding minimal redundant compliant modules. Such a simple design example is shown in Figure 2. The 1-DOF translational compliant mechanism shown in Figure 1a can be reconfigured into a symmetric 1-DOF translational compliant mechanism, as shown in Figure 2e having been reported in [29]. The specific design procedure can be seen in the following steps: (a) decomposing the compliant mechanism shown in Figure 1a into an MS, four BSs, and four two-beam compliant modules (TBCMs); (b) translating the compliant modules, labelled 2 and 3, to the positions shown in Figure 2b; (c) rotating the two compliant modules 180° to the new positions shown in Figure 2c; and (d) translating the two compliant modules to the positions shown in Figure 2d. Note that the two rearranged compliant modules should be linked to the MS with a RL. The deformation simulation of the symmetric 1-DOF translational compliant mechanism, under an actuation force, is shown in Figure 2f. It can be concluded that the symmetric 1-DOF translational compliant mechanism has minimal overconstraints under the single-axis actuation force, which is also a compact design compared to the traditional symmetric design (with more beams/overconstraints) as shown in [29].

Based on this position-space-based design concept of symmetric compliant mechanisms, this paper proposes a new symmetric spatial translational compliant parallel mechanism (XYZ CPM), which is reconfigured from a non-symmetric XYZ CPM by rearranging the compliant modules and adding minimal redundant compliant modules. The proposed symmetric XYZ CPM is less over-constrained compared with the traditional designed symmetric XYZ CPM. Additionally, the proposed symmetric XYZ CPM is also analyzed in terms of its motion performance, modelled, and manufactured. Note that the design of this symmetric XYZ CPM is one example to show the position-space-based design concept of symmetric compliant mechanisms. The proposed design concept can also be used to reconfigure other compliant mechanisms to symmetric ones as reported in [26,27,28,32].

The remainder of this paper is organized as follows. A non-symmetric XYZ CPM is reconfigured into a symmetric XYZ CPM in Section 2, followed by the analytical modelling of the symmetric XYZ CPM in Section 3. In Section 4, fabrication, assembly, and experimental testing are discussed. Finally, conclusions are presented in Section 5.

## 2. Design of a Symmetric XYZ CPM

**Figure 3:**
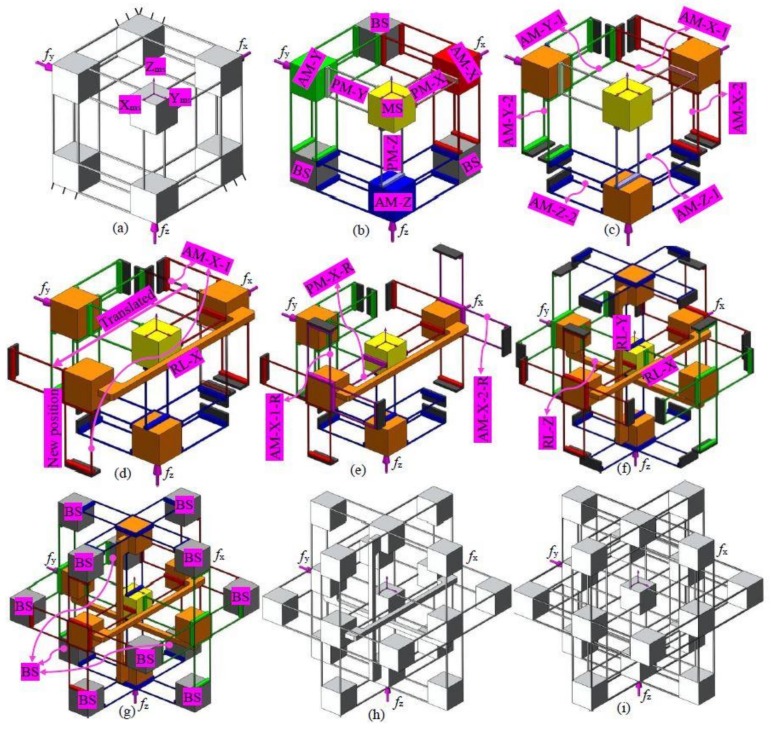
A symmetric XYZ compliant parallel mechanism (CPM) obtained via reconfiguring a non-symmetric XYZ CPM: (**a**) the original non-symmetric XYZ CPM [29]; (**b**) decomposition of the non-symmetric XYZ CPM; (**c**) further decomposition of the actuated compliant modules (AMs) of the non-symmetric XYZ CPM; (**d**) AM-X-1 translated to a new permitted position; (**e**) addition of redundant compliant modules (over-constraints); (**f**) reconfiguration of the legs associated with the translations along the Y_ms_- and Z_ms_-axes; (**g**) BS design; (**h**) resulting symmetric XYZ CPM; and (**i**) symmetric XYZ CPM designed by traditional approach, i.e., directly adding redundant compliant modules.

A non-symmetric and compact XYZ CPM was firstly conceived by Hao et al. [29]. It is an exactly-constrained design in its general type, with over-constraints in its three compositional actuated modules (Figure 3b). The non-symmetric XYZ CPM can provide decoupled translations along the X_ms_-, Y_ms_-, and Z_ms_-axes, but its parasitic rotations and cross-axis coupling are relatively large, which is not desired. Therefore, a symmetric XYZ CPM, with improved motion characteristics, is designed using the position space concept, based on the following steps.

(a) Decompose the non-symmetric XYZ CPM into rigid stages and compliant modules [26]. Figure 3b shows that the rigid stages are MS and BSs, and the compliant modules are actuated compliant modules (AMs: AM-X, AM-Y and AM-Z) and passive compliant modules (PMs: PM-X, PM-Y and PM-Z).
(b) Further decompose each of the AMs into two DTBCMs. Figure 3c illustrates that the AM-X is decomposed into two DTBCMs: AM-X-1 and AM-X-2; the AM-Y is decomposed into two DTBCMs: AM-Y-1 and AM-Y-2; the AM-Z is decomposed into two DTBCMs: AM-Z-1 and AM-Z-2.
(c) Reconfigure the AM-X by translating the AM-X-1 (within its position space) and its adjacent BSs along the X_ms_-axis, as shown in Figure 3d, so that the MS is located at the intermediate position between the AM-X-1 and the AM-X-2. As can be seen, a RL-X is needed to link the AM-X-1 and the AM-X-2.
(d) Add redundant compliant modules, AM-X-1-R and AM-X-2-R, as shown in Figure 3e, so that the AM-X is a mirror-symmetric compliant module about the MS. As studied in Section 1, a redundant copy of a compliant module can be added at any one position within the position space of the compliant module. Therefore, the positions of the AM-X-1-R and the AM-X-2-R should be within the position spaces of the AM-X-1 and the AM-X-2, respectively.
(e) Add a redundant PM, PM-X-R (Figure 3e), which is the reflection of the PM-X about the MS. In this case, the PM-X cannot be reconfigured to be symmetrical about the MS, so a redundant PM is added (the redundant PM is placed within the position space of the PM). By this step, the leg of the XYZ CPM associated with the X_ms_-axis translation is reconfigured.
(f) Reconfigure the other two legs of the XYZ CPM associated with the translations along the Y_ms_- and Z_ms_-axes, following the same reconfiguration process of the leg associated with the translation along the X_ms_-axis. The resulting design can be seen in Figure 3f.
(g) Re-design the BSs, as shown in Figure 3g.
(h) Combine all the rigid stages and compliant modules together (Figure 3h), which is the inverse process of decomposing the compliant mechanism. The symmetric XYZ CPM shown in Figure 3h is the resulting symmetric XYZ CPM.

Another symmetric XYZ CPM (Figure 3i) was designed using the traditional design approach by directly adding equal redundant compliant modules on the original non-symmetric XYZ CPM (Figure 3a). It can be seen that the traditional symmetric XYZ CPM shown in Figure 3i has more over-constraints compared with the new symmetric XYZ CPM shown in Figure 3h. More specifically, the quantity of the compliant beams in the symmetric XYZ CPM in Figure 3i has been reduced by 48 compared with the symmetric XYZ CPM in Figure 3h.

## 3. Nonlinear and Analytical Kinetostatic Modelling

### 3.1. Pre-Considerations

In order to provide more direct design insight, in this section, the relationships between the actuation forces and the given primary translations along the X_ms_-, Y_ms_-, and Z_ms_-axes are analytically modelled and verified by nonlinear finite element analysis (FEA) simulations. The nonlinear analytical models of the XYZ CPM can be used to estimate the actuation forces, and to predict the relationships between the actuation forces and the geometrical parameters, before conducting FEA simulations and experimental tests. Because the parasitic rotations (of AMs and MS) and the parasitic translations (of AMs) are much smaller than the associated primary translations (of AMs and MS), they can be ignored reasonably during the following analytical derivations of primary forces/motions [23,31], and their analytical (closed-form) models are not considered in this paper. The parasitic motions are also not modelled in this paper, because they are much smaller than the primary motions.

Additionally, in this paper, all the beams are the same with uniform square cross sections. Displacements and lengths are normalized by the beam length *L*, forces are normalized by *EI*/*L*^2^, and moments are normalized by *EI*/*L*. Here, *E* is the Young’s modulus of the material and *I* is the second moment inertia of cross-section area of the uniform beam [23].

### 3.2. Closed-Form Modelling

**Figure 4:**
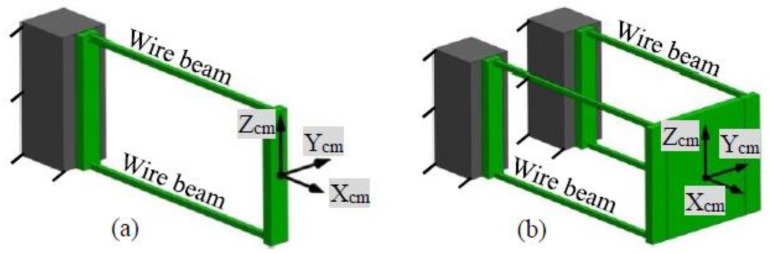
Compliant modules: (**a**) A two-beam compliant module (TBCM) and its coordinate system and (**b**) a four-beam compliant module and its coordinate system.

Each PM of the symmetric XYZ CPM can be referred to as a two-beam compliant module (TBCM) as shown in Figure 4a, and each AM of the symmetric XYZ CPM can be regarded as a combination of TBCMs. Therefore, the motions of the symmetric XYZ CPM are performed through the deformation of the TBCMs, so the modelling of the TBCM is carried out before modelling the symmetric XYZ CPM. Note that all the cubes as rigid stages are identical.

The reaction forces produced by the deformation of the four-beam compliant module, as shown in Figure 4b, is presented in [18], which was first reported in [33]. The four-beam compliant module is a combination of two identical TBCMs shown in Figure 4a. If ignoring the rotations of the TBCM, the reaction forces produced by the deformation of the TBCM along its X_cm_-, Y_cm_-, and Z_cm_-axes are 0.5 times of the reaction forces produced by the deformation of the four-beam compliant module along its X_cm_-, Y_cm_-, and Z_cm_-axes, respectively. Therefore, the reaction forces produced by the deformation of the TBCM along its X_cm_-, Y_cm_-, and Z_cm_-axes can be obtained by using the results of the reaction forces of the TBCM derived in [18], which are shown in Equations (1)–(3), respectively.

(1) $$\zeta_{\mathrm{cm}\text{-}\mathrm{tx}}=-\frac{840\left(5\xi_{\mathrm{cm}\text{-}\mathrm{tx}}+3\left(\xi_{\mathrm{cm}\text{-}\mathrm{ty}}^{2}+\xi_{\mathrm{cm}\text{-}\mathrm{tz}}^{2}\right)\right)}{175t^{2}+3\xi_{\mathrm{cm}\text{-}\mathrm{ty}}^{2}+3\xi_{\mathrm{cm}\text{-}\mathrm{tz}}^{2}}$$

(2) $$\zeta_{\mathrm{cm}\text{-}\mathrm{ty}}=-\frac{24\xi_{\mathrm{cm}\text{-}\mathrm{ty}}\left(175t^{2}+210\xi_{\mathrm{cm}\text{-}\mathrm{tx}}+129\xi_{\mathrm{cm}\text{-}\mathrm{ty}}^{2}+129\xi_{\mathrm{cm}\text{-}\mathrm{tz}}^{2}\right)}{175t^{2}+3\xi_{\mathrm{cm}\text{-}\mathrm{ty}}^{2}+3\xi_{\mathrm{cm}\text{-}\mathrm{tz}}^{2}}$$

(3) $$\zeta_{\mathrm{cm}\text{-}\mathrm{tz}}=-\frac{24\xi_{\mathrm{cm}\text{-}\mathrm{tz}}\left(175t^{2}+210\xi_{\mathrm{cm}\text{-}\mathrm{tx}}+129\xi_{\mathrm{cm}\text{-}\mathrm{ty}}^{2}+129\xi_{\mathrm{cm}\text{-}\mathrm{tz}}^{2}\right)}{175t^{2}+3\xi_{\mathrm{cm}\text{-}\mathrm{ty}}^{2}+3\xi_{\mathrm{cm}\text{-}\mathrm{tz}}^{2}}$$

where *t* is the thickness of the beam. *ξ*_cm-tx_, *ξ*_cm-ty_, and *ξ*_cm-tz_ are the primary translational displacements of the TBCM. *ζ*_cm-tx_, *ζ*_cm-ty_, and *ζ*_cm-tz_ are the reaction forces along the X_cm_-, Y_cm_-, and Z_cm_-axes, respectively, produced by the TBCM due to the deformation.

**Figure 5:**
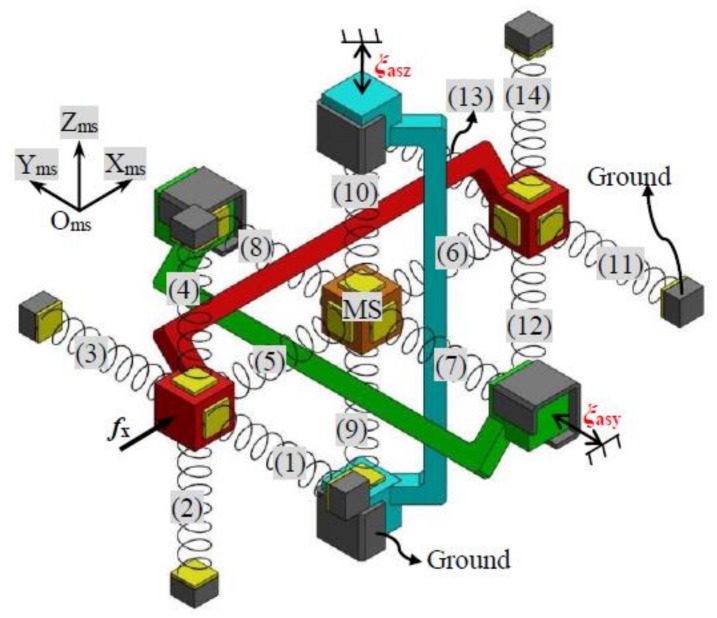
Simplified spring model of the symmetric XYZ CPM with illustrative force actuation along the X-axis (RL-X is red in color, RL-Y is green in color, and RL-Z is blue in color).

A TBCM can be regarded as a three-dimensional translational spring. The complete symmetric XYZ CPM can be modelled based on the analytical model of the three-dimensional translational spring. The motion performance of the symmetric XYZ CPM along the X_ms_-, Y_ms_-, and Z_ms_-axes are isotropic (O_ms_-X_ms_Y_ms_Z_ms_ is the global coordinate system in this section). Therefore, only the primary translations along one of the three directions need to be studied. In this paper, the derivation of the force-displacement relationship, associated with only the translations along the X_ms_-axis, is detailed. Given any primary displacements, *ξ*_asy_ and *ξ*_asz_, of the RL-Y and RL-Z, respectively, the XYZ CPM can be simplified to the model shown in Figure 5 if only the force-displacement relationship in X_ms_-axis is considered.

Let the lost motions along the X_ms_-, Y_ms_-, and Z_ms_-axes be *δ*_x_, *δ*_y_, and *δ*_z_, respectively, which can be written as below

(4) $$\delta_{x}=\xi_{\mathrm{asx}}-\xi_{\mathrm{msx}},\;\delta_{y}=\xi_{\mathrm{asy}}-\xi_{\mathrm{msy}}\;\mathrm{and}\;\delta_{z}=\xi_{\mathrm{asz}}-\xi_{\mathrm{msz}}$$

where *ξ*_msx_, *ξ*_msy_ and *ξ*_msz_ are the primary translations of the MS along the X_ms_-, Y_ms_-, and Z_ms_-axes, respectively. The primary translations of the RL-X, the RL-Y, and the RL-Z are represented by *ξ*_asx_, *ξ*_asy_, and *ξ*_asz_, respectively. The model, as shown in Figure 5, contains 14 TBCMs in each axis, which are termed as TBCM-1 to TBCM-14. If all the parasitic rotations and parasitic translations of the symmetric XYZ CPM are ignored, the deformation displacements of each of the TBCMs can be obtained easily according to the primary translations and lost motions. Additionally, the reaction forces of the TBCMs can also be calculated based on Equations (1)–(3). Taking the TBCM-1 as an example, the TBCM-1 is linked to the RL-X, so the deformation displacements of the TBCM-1 can be derived from the motion displacements of the RL-X. If ignoring all the parasitic rotations and parasitic translations of the RL-X, the deformation displacements of the TBCM-1 equal to *ξ*_asx_, zero, and zero along the X_ms_-, Y_ms_-, and Z_ms_-axes, respectively. Therefore, the reaction force, *ζ*_a_, of the TBCM-1 along the X_ms_-axis can be obtained, as shown in Equation (5), by substituting the deformation displacements of the TBCM-1 into Equation (2) or (3). Note that when substituting the deformation displacements into Equation (2), *ξ*_cm-tx_, *ξ*_cm-ty_, and *ξ*_cm-tz_ in Equation (2) equal to zero, *ξ*_asx_, and zero, respectively; when substituting the deformation displacements into Equation (3), *ξ*_cm-tx_, *ξ*_cm-ty_, and *ξ*_cm-tz_ in Equation (3) equal to zero, zero, and *ξ*_asx_, respectively. Similarly, the reaction force of the TBCM-2, TBCM-3, TBCM-4, TBCM-11, TBCM-12, TBCM-13, or TBCM-14, to the RL-X along the X_ms_-axis, can also be obtained as shown in Equation (5). The reaction forces of the TBCM-5, TBCM-6, TBCM-7, TBCM-8, TBCM-9, and TBCM-10, to the MS along the X_ms_-axis, can be derived from Equations (6) to (11), respectively, which are represented as *ζ*_b_, *ζ*_c_, *ζ*_d_, *ζ*_e_, *ζ*_f_, and *ζ*_g_, respectively, as below.

(5) $$\zeta_{a}=-\frac{24\xi_{\mathrm{asx}}\left(129\xi_{\mathrm{asx}}^{2}+175t^{2}\right)}{3\xi_{\mathrm{asx}}^{2}+175t^{2}}$$

(6) $$\zeta_{b}=\frac{840\left(3\left(\xi_{\mathrm{msy}}^{2}+\xi_{\mathrm{msz}}^{2}\right)-5\delta_{x}\right)}{3\xi_{\mathrm{msy}}^{2}+3\xi_{\mathrm{msz}}^{2}+175t^{2}}$$

(7) $$\zeta_{c}=-\frac{840\left(3\left(\xi_{\mathrm{msy}}^{2}+\xi_{\mathrm{msz}}^{2}\right)+5\delta_{x}\right)}{3\xi_{\mathrm{msy}}^{2}+3\xi_{\mathrm{msz}}^{2}+175t^{2}}$$

(8) $$\zeta_{d}=-\frac{24\xi_{\mathrm{msx}}\left(-210\delta_{y}+129\xi_{\mathrm{msx}}^{2}+129\xi_{\mathrm{msz}}^{2}+175t^{2}\right)}{3\xi_{\mathrm{msx}}^{2}+3\xi_{\mathrm{msz}}^{2}+175t^{2}}$$

(9) $$\zeta_{e}=-\frac{24\xi_{\mathrm{msx}}\left(210\delta_{y}+129\xi_{\mathrm{msx}}^{2}+129\xi_{\mathrm{msz}}^{2}+175t^{2}\right)}{3\xi_{\mathrm{msx}}^{2}+3\xi_{\mathrm{msz}}^{2}+175t^{2}}$$

(10) $$\zeta_{f}=-\frac{24\xi_{\mathrm{msx}}\left(-210\delta_{z}+129\xi_{\mathrm{msy}}^{2}+129\xi_{\mathrm{msx}}^{2}+175t^{2}\right)}{3\xi_{\mathrm{msy}}^{2}+3\xi_{\mathrm{msx}}^{2}+175t^{2}}$$

(11) $$\zeta_{g}=-\frac{24\xi_{\mathrm{msx}}\left(210\delta_{z}+129\xi_{\mathrm{msy}}^{2}+129\xi_{\mathrm{msx}}^{2}+175t^{2}\right)}{3\xi_{\mathrm{msy}}^{2}+3\xi_{\mathrm{msx}}^{2}+175t^{2}}$$

When the MS is in static equilibrium, all the reaction forces on the MS along the X_ms_-axis should be balanced, so Equation (12) can be obtained [34]. When substituting Equations (6)–(11) into Equation (12), Equation (13) for the lost motion along the X_ms_-axis can be derived. Furthermore, the actuation force, *f*_x_, should be equal to the sum of the reaction forces of all the TBCMs except TBCM-5 and TBCM-6, along the X_ms_-axis. Therefore, the relationship between the actuation force *f*_x_ and the primary translations of the MS can be obtained, as shown in Equation (14). Similarly, the force-displacement relationships associated with the actuation forces, *f*_y_ and *f*_z_, can be derived, as shown in Equations (14)–(16). Note that the actuation forces, *f*_y_ and *f*_z_, are applied on the RL-Y and RL-Z, respectively.

(12) $$\zeta_{b}+\zeta_{c}+\zeta_{d}+\zeta_{e}+\zeta_{f}+\zeta_{g}=0$$

(13) $$\begin{array}{l} \delta_{x}=\frac{6\xi_{\mathrm{msx}}^{3}\xi_{\mathrm{msyz}}\left(129\xi_{\mathrm{msy}}^{2}+129\xi_{\mathrm{msz}}^{2}+7700t^{2}\right)}{175\xi_{\mathrm{msxy}}\xi_{\mathrm{msxz}}}\;+\frac{6\xi_{\mathrm{msx}}\xi_{\mathrm{msy}}^{2}\xi_{\mathrm{msyz}}\left(129\xi_{\mathrm{msz}}^{2}+3850t^{2}\right)}{175\xi_{\mathrm{msxy}}\xi_{\mathrm{msxz}}}+\;\frac{774\xi_{\mathrm{msx}}^{5}\xi_{\mathrm{msyz}}}{175\xi_{\mathrm{msxy}}\xi_{\mathrm{msxz}}}\\ \;+\;\frac{2\xi_{\mathrm{msx}}\xi_{\mathrm{msyz}}\left(66t^{2}\xi_{\mathrm{msz}}^{2}+175t^{4}\right)}{\xi_{\mathrm{msxy}}\xi_{\mathrm{msxz}}}\\ \mathrm{where}\;\xi_{\mathrm{msxy}}=3\xi_{\mathrm{msx}}^{2}+3\xi_{\mathrm{msy}}^{2}+175t^{2},\;\xi_{\mathrm{msxz}}=3\xi_{\mathrm{msx}}^{2}+3\xi_{\mathrm{msz}}^{2}+175t^{2},\;\xi_{\mathrm{msyz}}=3\xi_{\mathrm{msy}}^{2}+3\xi_{\mathrm{msz}}^{2}+175t^{2} \end{array}$$

(14) $$f_{x}=\frac{48\xi_{\mathrm{msx}}\left(129\xi_{\mathrm{msx}}^{2}+129\xi_{\mathrm{msz}}^{2}+175t^{2}\right)}{3\xi_{\mathrm{msx}}^{2}+3\xi_{\mathrm{msz}}^{2}+175t^{2}}\;+\frac{48\xi_{\mathrm{msx}}\left(129\xi_{\mathrm{msx}}^{2}+129\xi_{\mathrm{msy}}^{2}+175t^{2}\right)}{3\xi_{\mathrm{msx}}^{2}+3\xi_{\mathrm{msy}}^{2}+175t^{2}}\;+\frac{192\xi_{\mathrm{msx}}\left(129\xi_{\mathrm{msx}}^{2}+175t^{2}\right)}{3\xi_{\mathrm{msx}}^{2}+175t^{2}}$$

(15) $$f_{y}=\frac{48\xi_{\mathrm{msy}}\left(129\xi_{\mathrm{msy}}^{2}+129\xi_{\mathrm{msz}}^{2}+175t^{2}\right)}{3\xi_{\mathrm{msy}}^{2}+3\xi_{\mathrm{msz}}^{2}+175t^{2}}\;+\frac{48\xi_{\mathrm{msy}}\left(129\xi_{\mathrm{msx}}^{2}+129\xi_{\mathrm{msy}}^{2}+175t^{2}\right)}{3\xi_{\mathrm{msx}}^{2}+3\xi_{\mathrm{msy}}^{2}+175t^{2}}+\frac{192\xi_{\mathrm{msy}}\left(129\xi_{\mathrm{msy}}^{2}+175t^{2}\right)}{3\xi_{\mathrm{msy}}^{2}+175t^{2}}$$

(16) $$f_{z}=\frac{48\xi_{\mathrm{msz}}\left(129\xi_{\mathrm{msx}}^{2}+129\xi_{\mathrm{msz}}^{2}+175t^{2}\right)}{3\xi_{\mathrm{msx}}^{2}+3\xi_{\mathrm{msz}}^{2}+175t^{2}}+\frac{48\xi_{\mathrm{msz}}\left(129\xi_{\mathrm{msz}}^{2}+129\xi_{\mathrm{msy}}^{2}+175t^{2}\right)}{3\xi_{\mathrm{msz}}^{2}+3\xi_{\mathrm{msy}}^{2}+175t^{2}}+\frac{192\xi_{\mathrm{msz}}\left(129\xi_{\mathrm{msz}}^{2}+175t^{2}\right)}{3\xi_{\mathrm{msz}}^{2}+175t^{2}}$$

Using Equations (14)–(16), the actuation forces, *f*_x_, *f*_y_, and *f*_z_, can be obtained when specific translational displacements of the MS (output motions), *ξ*_msx_, *ξ*_msy_, and *ξ*_msz_, are required. Furthermore, the primary translational displacements of the RLs (input motions), *ξ*_asx_, *ξ*_asy_, and *ξ*_asz_, can also be obtained according to Equation (4).

### 3.3. Quantitative Analysis and Comparisons

In this section, FEA simulations are carried out to verify the analytical models derived in Section 4.2 and to analyze parasitic motion characteristics of the proposed symmetric XYZ CPM (shown in Figure 3h). Additionally, the motion characteristics of the non-symmetric XYZ CPM (shown in Figure 3a) are also analyzed by FEA simulations. Therefore, quantitative comparisons between the symmetric and non-symmetric XYZ CPMs in terms of their motion characteristics can be obtained in this section. Note that the commercial software, COMSOL MULTIPHYSICS (version 5.0, COMSOL Inc., Cambridge, UK), is selected for the nonlinear FEA simulations. We used the 10-node tetrahedral meshing element and fine meshing techniques with default element size parameters (14 mm maximum element size, 1.75 mm minimum element size, 1.45 maximum element growth rate, 0.5 curvature factor, and 0.6 resolution of narrow regions). In COMSOL MULTIPHYSICS, there are nine different levels of meshing techniques from extremely coarse to extremely fine. The fine meshing technique is the fourth best one. Based on the selected meshing technique, the simulation model is meshed automatically by the software. The meshed model shows that the beams have much smaller meshing elements than the stages. The meshing settings in this paper are the same as those in [18].

**Figure 6:**
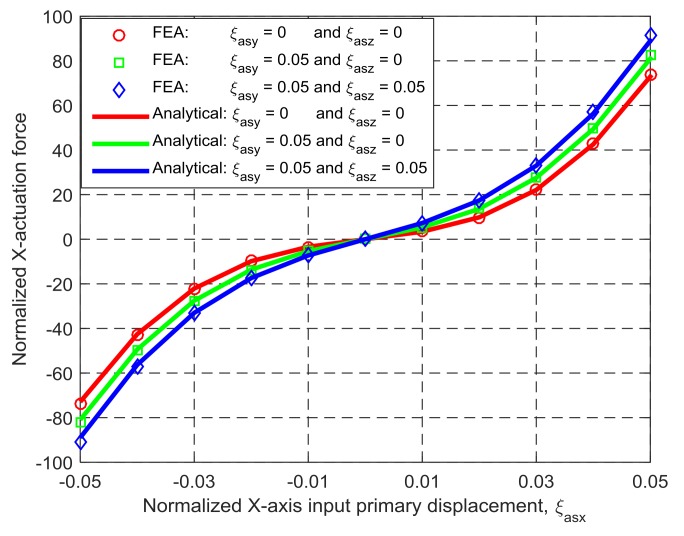
Comparison between the analytical results and the finite element analysis (FEA) results in terms of the force-displacement relationship.

The derived nonlinear analytical models in Section 3.2 are applicable for any geometrical dimension and material. For a case study in this section, the length and the thickness of the identical wire beams are assigned to be 50 mm and 1 mm, the spanning size of the TBCMs is 25 mm, Young’s modulus of material is 69 GPa, and Poisson’s ratio of material is 0.33. The analytical results and the FEA results, in terms of the X_ms_-axis actuation force, can be seen in Figure 6 under the following actuation displacement conditions: (a) *ξ*_asx_ varies from −0.05 to +0.05, *ξ*_asy_ = 0, and *ξ*_asz_ = 0; (b) *ξ*_asx_ varies from −0.05 to +0.05, *ξ*_asy_ = 0.05, and *ξ*_asz_ = 0; and (c) *ξ*_asx_ varies from −0.05 to +0.05, *ξ*_asy_ = 0.05, and *ξ*_asz_ = 0.05. Each of the actuation displacements is added to the simulation model by pre-setting the displacement of the center point of the outside surface of the actuated rigid stage (the point is also the one that the actuation force is applied on, as shown in Figure 5). The directions of the actuation displacements are keep the same.

Figure 6 shows that the analytical results and the FEA results of the X_ms_-axis actuation force match very well, with less than 2.58% difference. The difference rises with the increase of the cross-axis input displacements.

**Figure 7:**
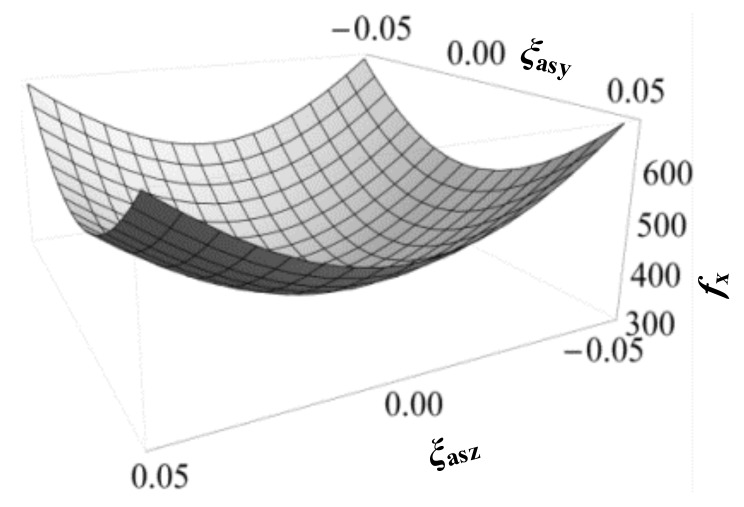
Variation of actuation force, *f*_x_, with *ξ*_asy_ and *ξ*_asz_, when *ξ*_asx_ = 0.

Based on the analytical models of the symmetric XYZ CPM, the X_ms_-axis actuation force is further analyzed, which can be seen in Figure 7. It can be seen that the actuation force, *f*_x_, increases with the increase of *ξ*_asy_ and *ξ*_asz_. It can also be derived that the actuation stiffness increases along the X_ms_-axis with the increase of the primary translations along the other directions.

**Figure 8:**
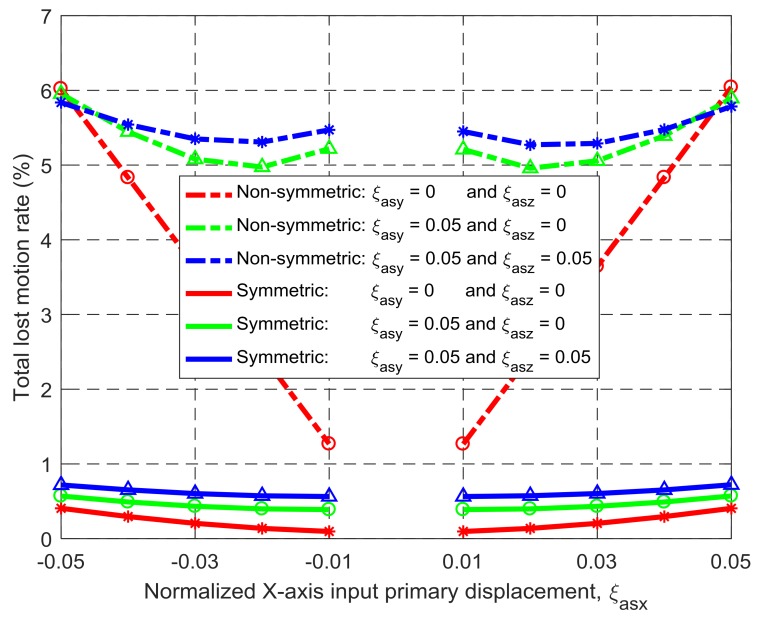
Comparison of lost motions between the symmetric and non-symmetric XYZ CPMs shown in Figure 3a,h (Symbols, ‘*’, ‘△’ and ‘○’, in this figure are data points).

**Figure 9:**
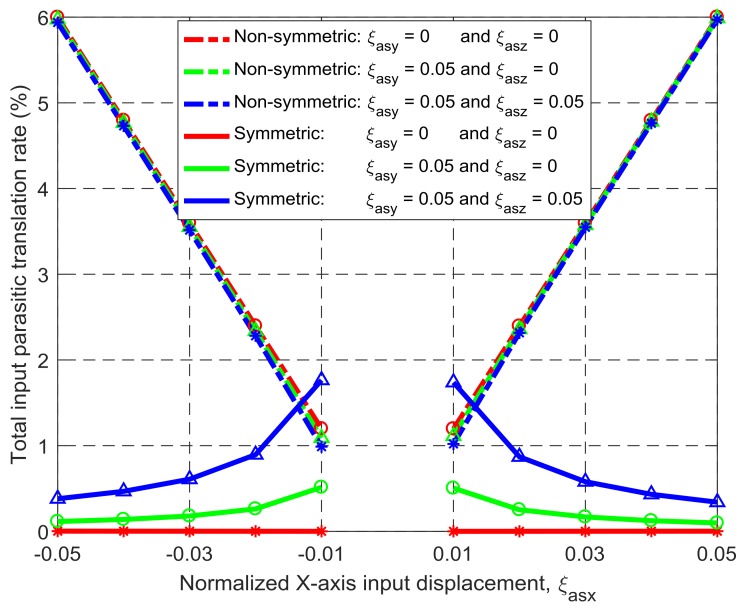
Comparison of input parasitic translations between the symmetric and non-symmetric XYZ CPMs shown in Figure 3a,h (Symbols, ‘*’, ‘△’ and ‘○’, in this figure are data points).

**Figure 10:**
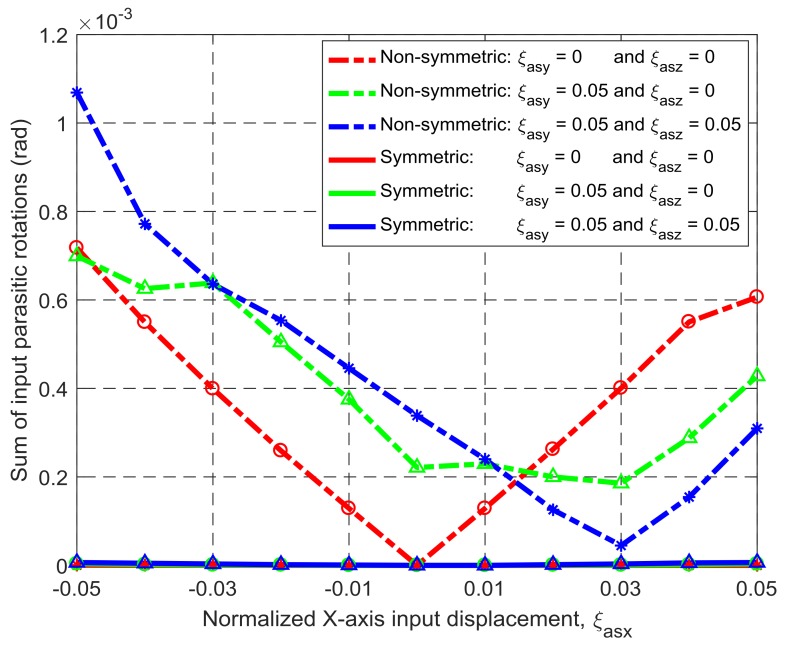
Comparison of input parasitic rotations between the symmetric and non-symmetric XYZ CPMs shown in Figure 3a,h (Symbols, ‘*’, ‘△’ and ‘○’, in this figure are data points).

**Figure 11:**
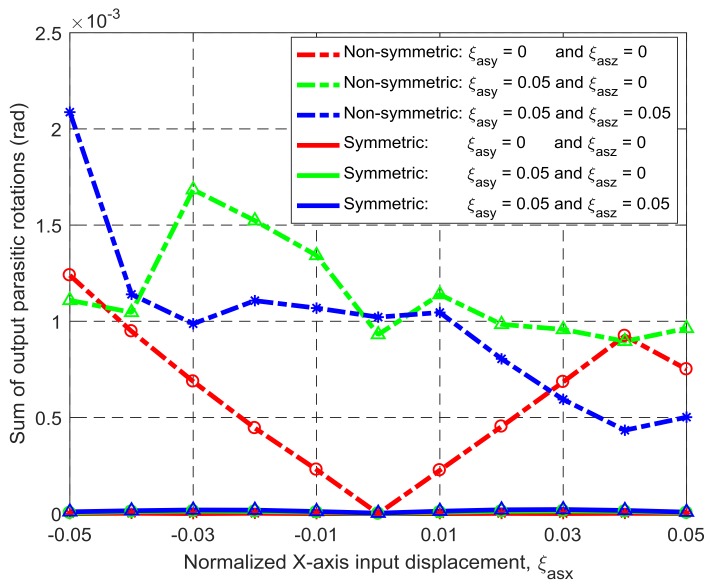
Comparison of output parasitic rotations between the symmetric and non-symmetric XYZ CPMs shown in Figure 3a,h (Symbols, ‘*’, ‘△’ and ‘○’, in this figure are data points).

**Figure 12:**
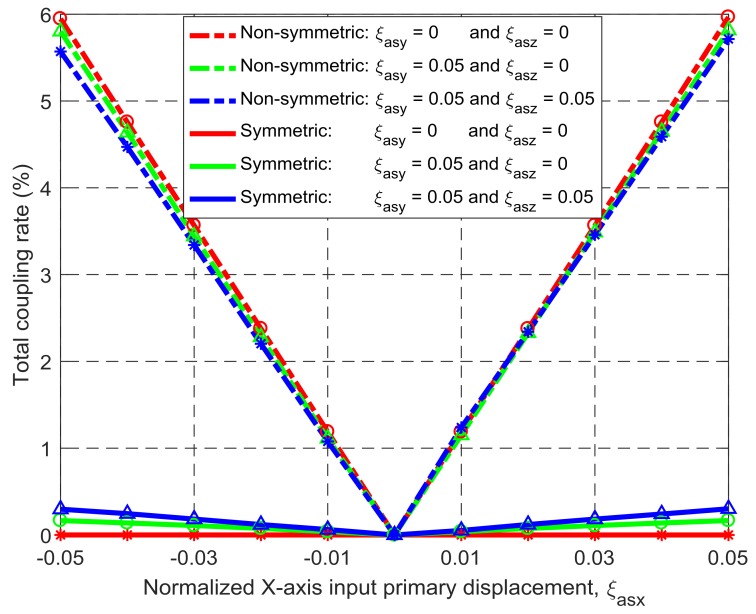
Coupling comparison between the symmetric and non-symmetric XYZ CPMs shown in Figure 3a,h (Symbols, ‘*’, ‘△’ and ‘○’, in this figure are data points).

Under the same simulation conditions, the motion characteristics of the non-symmetric XYZ CPM (shown in Figure 3a) are also derived using FEA simulations. Therefore, comparisons between the symmetric and non-symmetric XYZ CPMs (shown in Figure 3a,h) can be made in terms of their lost motions, parasitic motions (input parasitic translations, input parasitic rotations, and output parasitic rotations), and cross-axis coupling. Note that lost motion can be reflected by lost motion rate, parasitic motion can be reflected by parasitic motion rate, and cross-axis coupling can be reflected by cross-axis coupling rate. Herein, the ratio of a lost motion displacement to the associated primary displacement refers to the lost motion rate, the ratio of a parasitic displacement to the associated primary displacement is the parasitic motion rate, and the ratio of a cross-axis coupling displacement to the associated primary displacement defines the coupling rate. The comparison results are shown in Figure 8, Figure 9, Figure 10, Figure 11 and Figure 12. It can be observed that: (a) the lost motion rate of the symmetric design is approximately 10 times lower than that of the non-symmetric design, as shown in Figure 8; (b) the input parasitic translations of the symmetric design is also significantly smaller than that of the non-symmetric design in most cases, as shown in Figure 9; (c) the symmetric design has tiny input and output parasitic rotations compared with the non-symmetric design, as shown in Figure 10 and Figure 11; and (d) Figure 12 shows that the non-symmetric design has much larger (up to approximately 30 times) cross-axis coupling compared with the symmetric design. Therefore, the symmetric XYZ CPM has better motion performance, ensuring its convincing application in high-precision positioning. However, the geometrical structure of the symmetric XYZ CPM is complex for manufacturing compared with the non-symmetric XYZ CPM. Therefore, it was necessary to study the fabrication of the symmetric XYZ CPM in the following section.

## 4. Fabrication and Experimental Tests

### 4.1. Fabrication Consideration

The designed symmetric XYZ CPM shown in Figure 3h can provide translations along the X_ms_-, Y_ms_-, and Z_ms_-axes with desired motion characteristics. However, the MS of the symmetric XYZ CPM is located at the center of the whole structure, and the symmetric XYZ CPM cannot be fabricated monolithically. Therefore, a practical design of the symmetric XYZ CPM is figured out in this section, and a prototype of the practical design is also presented.

**Figure 13:**
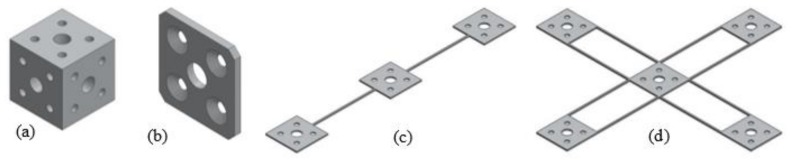
Main assembling components: (**a**) rigid cube; (**b**) rigid washer; (**c**) compliant passive compliant modules (PM) beam; and (**d**) compliant AM beam.

**Figure 14:**
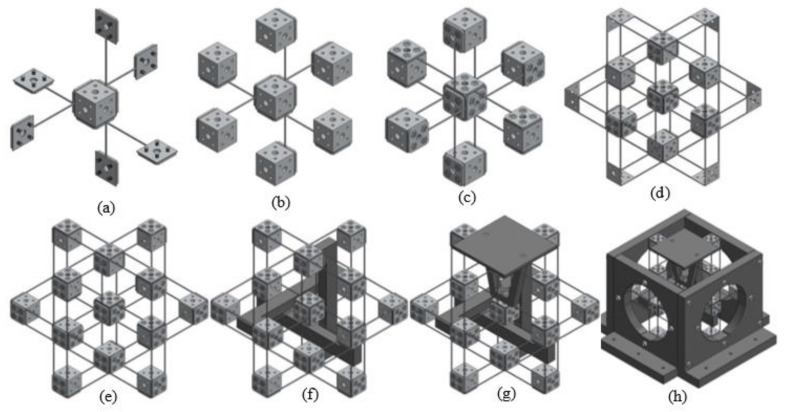
Assembling demonstration of the practical design: (**a**–**e**) assembling of rigid cubes, rigid washers, compliant PM beams, and compliant AM beams; (**f**) assembling of three RLs; (**g**) assembling of output platform; and (**h**) assembling of supporting seat.

The XYZ CPM cannot be fabricated monolithically, so assembling components are designed as shown in Figure 13. The components, with mounting holes, are fabricated using a computer numeric control milling machine. These components are assembled together using screws, as shown in Figure 14a–e. Figure 14f–h show that three RLs, one output platform, and one supporting seat are assembled to the system shown in Figure 9e. The external output platform is rigidly connected to the MS. Therefore, the motion of the MS can be transmitted to the output platform, i.e., the output platform can translate in the three orthogonal directions under the actuation of the three actuators. The rigid components and the compliant components can be fabricated using different materials for better performance (for example, the AM stages can be the material with low thermal conductivity so that the heat from a voice coil actuator is hard to transfer to the wire beams). Additionally, if one of the components is broken, it is easy to replace it with a new one. However, the assembling can reduce the system stiffness, hence it may affect the dynamic performance (for example decreasing the natural frequency of the system) and motion accuracy. In order to reduce the influences of the assembling, large pre-stressing forces were applied to the fastened parts.

### 4.2. Prototype Testing

**Figure 15:**
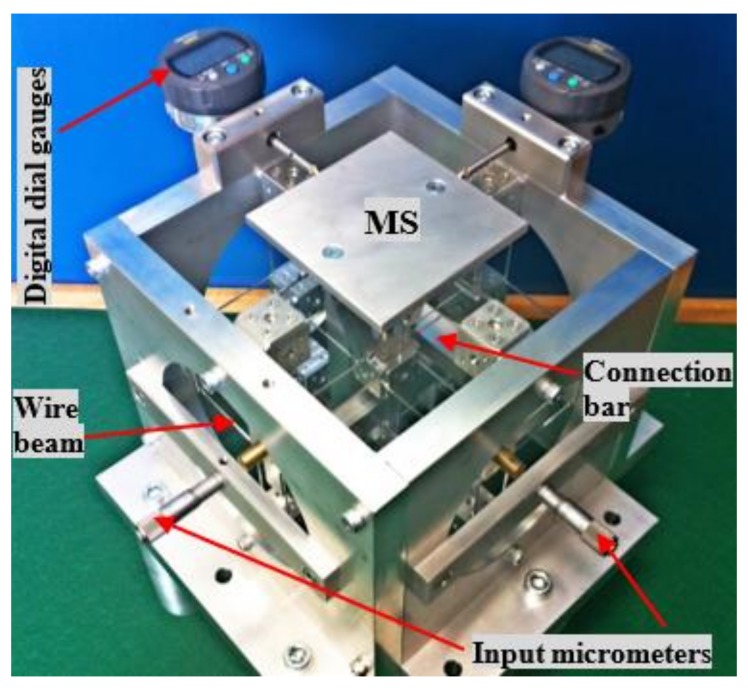
A prototype of the practical design.

A prototype of the XYZ CPM with three micrometers as displacement inputs is obtained based on the practical design, which is shown in Figure 15. All components of the prototype are made of Aluminum 6061 (Young’s modulus is about 69 GPa and Poisson’s ratio is about 0.33). The output displacements of the MS along the X_ms_- and Y_ms_-axes are separately measured by two digital indicators. Each indicator has a resolution of 1 µm and a very low spring force of 0.4–0.7 N (Digimatic Indicators, Mitutoyo Corporation, Kawasaki, Japan). The input displacements along the X_ms_-, Y_ms_-, and Z_ms_-axes are actuated by three micrometers with a resolution of 1 µm. The gravity (approximately 1.36 N) of the mobile parts of the prototype can affect slightly the input forces, especially influencing the input force along the Z_ms_-axis direction. The gravity can also have very small influence on the output displacement along the Z_ms_-axis, but it cannot affect any of the input displacements because the input displacements are directly controlled by three micrometers. Similarly, the small spring forces (0.4–0.7 N) of the digital indicators have trivial influence on the output displacements along the X_ms_- and Y_ms_-axes, but no influence on the input displacements. Because the gravity and the indicators’ spring forces are very small, their effects on the output displacements are ignored in this paper. In addition, because the parasitic motions of the input stages are very tiny, as shown earlier due to the symmetric design, the coupling errors among the input stages/micrometers can also be ignored for this experimental test.

**Figure 16:**
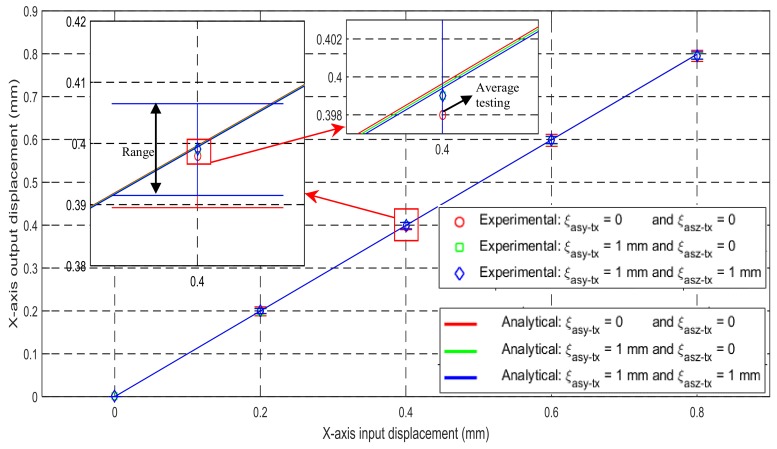
Relationship between the input displacement and the output displacement along the X_ms_-axis.

The relationships of the input and output translations along the X-axis are experimentally tested on the prototype. The maximum difference between the experimental results and the analytical results is less than 0.52%, as shown in Figure 16. Note that each experimental displacement value marked in Figure 16 is the average of three displacements obtained from three repeated experimental tests (the range of experimental data corresponding to each average with the same color is labelled). The difference between the experiment and analytical models may arise from machining errors, assembly errors, the gravity of the mobile parts, and/or the micrometer coupling errors, as well as the undesired deformation of connection bars. If the rigid cubes and the RLs are made of a lighter material with higher Young’s modulus, the experimental results can match the analytical results better. It can also be observed from the experimental results that the cross-axis coupling rate and the lost motion rate are less than 0.13% and 0.63%, respectively. Similar to the verification by the FEA simulations, the experimental testing can confirm: (1) the reasonably high accuracy of the analytical models obtained in Section 3.2; (2) the decreased lost motions; and (3) the small influences of the parasitic motions and cross-axis coupling on the motion accuracy.

In this paper, only the prototype’s micro-scale resolution is verified by the micrometers and digital indicators. The prototype’s nano-positioning precision, with an accuracy of 5 nm, repeatability of 35 nm, and resolution of 30 nm, has been reported in the authors’ other work [35,36].

## 5. Conclusions

This paper proposes a new position-space-based approach for reconfiguring non-symmetric compliant mechanisms into symmetric compliant mechanisms. Compared with existing designed symmetric compliant mechanisms, the symmetric compliant mechanisms designed using this position-space-based approach are less over-constrained due to the fact that reduced redundant compliant modules are added, and have better reliability as fewer beams are employed. Based on the position-space-based approach, a non-symmetric XYZ CPM is reconfigured into a symmetric XYZ CPM in this paper. Compared with the original non-symmetric XYZ CPM (Figure 3a), the designed symmetric XYZ CPM has much better performance characteristics including minimized parasitic motions, dropped cross-axis coupling, and reduced lost motions, which is verified using FEA simulations (Figure 8, Figure 9, Figure 10, Figure 11 and Figure 12). Compared with the traditional symmetric mechanism design (Figure 3i), the new symmetric XYZ CPM also has advantages such as having fewer overconstraints, smaller actuation stiffness, and a less complex structure, due to using a smaller number of compliant beams.

## Nomenclature

DOF	degree(s) of freedom
DOC	degree(s) of constraint
FEA	finite element analysis
CPM	compliant parallel mechanism
MS	motion stage
BS	base stage
AM	actuated compliant module
PM	passive compliant module
RL	rigid link
TBCM	two-beam compliant module
DTBCM	double-two-beam compliant module
